# Ionomer-directed reaction pathway for CO_2_ electroreduction

**DOI:** 10.1093/nsr/nwag364

**Published:** 2026-06-12

**Authors:** Yaoyu Yin, Xinchen Kang, Buxing Han

**Affiliations:** Beijing National Laboratory for Molecular Sciences, CAS Laboratory of Colloid and Interface and Thermodynamics, CAS Research/Education Centre for Excellence in Molecular Sciences, Centre for Carbon Neutral Chemistry, Institute of Chemistry, Chinese Academy of Sciences, Beijing 100190, China; School of Chemistry and Chemical Engineering, University of Chinese Academy of Sciences, Beijing 100049, China; Beijing National Laboratory for Molecular Sciences, CAS Laboratory of Colloid and Interface and Thermodynamics, CAS Research/Education Centre for Excellence in Molecular Sciences, Centre for Carbon Neutral Chemistry, Institute of Chemistry, Chinese Academy of Sciences, Beijing 100190, China; School of Chemistry and Chemical Engineering, University of Chinese Academy of Sciences, Beijing 100049, China; Beijing National Laboratory for Molecular Sciences, CAS Laboratory of Colloid and Interface and Thermodynamics, CAS Research/Education Centre for Excellence in Molecular Sciences, Centre for Carbon Neutral Chemistry, Institute of Chemistry, Chinese Academy of Sciences, Beijing 100190, China; School of Chemistry and Chemical Engineering, University of Chinese Academy of Sciences, Beijing 100049, China

**Keywords:** CO_2_ electroreduction, ionomer, interfacial microenvironment, electrode

## Abstract

Electrochemical CO_2_ reduction reaction (CO_2_RR) is a sustainable method of converting carbon emissions into valuable fuels and chemicals; however, its selectivity and stability are governed by the catalyst itself and the local microenvironment within the catalyst layer. Ionomers, once considered to be passive binders, have emerged as key regulators of this microenvironment. However, the ionomer content in the catalyst layer is very low, because of which its effect on the CO_2_RR performance is often ignored. This review aims to address this gap. First, the structural diversity of proton- and anion-exchange ionomers is discussed, and their distinct physicochemical properties, which are critical for shaping ion transport and interfacial reactions, are described. Next, the effects of solvent-controlled dispersion and film formation on the spatial distribution and morphology of ionomers in the catalyst layers and the subsequent effects on mass transport and local hydration are discussed. Based on this foundation, the control of CO_2_RR pathways via the ionomer configuration is discussed, specifically considering the coupled effects on reactant diffusion, intermediate adsorption energetics, and interfacial hydrogen bond networks. Finally, the current challenges and future opportunities are summarized, with an emphasis on nanoscale characterization, ionomer durability, and the co-design of catalysts and ionomers. By elucidating the molecular-level connections between ionomer structure, dispersion, and catalytic function, this review establishes design principles that can guide the development of next-generation electrodes and electrolyzers for efficient, selective, and durable CO_2_ conversion. Moreover, as demonstrated in this review, these principles are broadly applicable to a wide range of other electrocatalytic reactions.

## Teaser

This review reveals that ionomers, beyond serving as binders, regulate local reaction environments to steer CO_2_ electroreduction along more efficient and selective reaction pathways.

## INTRODUCTION

Since the industrial revolution, large-scale deforestation and excessive fossil fuel consumption have drastically increased the atmospheric CO_2_ concentration from the pre-industrial level of ∼277 ppm to more than 400 ppm, and it is expected to reach 600 ppm by 2100 [1–4]. This concentration greatly exceeds the recognized safety threshold of 350 ppm, and it has been directly linked to global warming, desertification, and biodiversity loss. Significant attention has been dedicated to non-conversion and direct chemical conversion of CO_2_ amid efforts to mitigate these environmental challenges. Among various strategies, including biological, thermochemical, electrochemical, and photochemical approaches, the electrochemical CO_2_ reduction reaction (CO_2_RR) has attracted particular attention [5–8]. Unlike other processes, the CO_2_RR can be driven under ambient conditions in modular and scalable electrochemical systems. Hence, it offers a route for converting stable CO_2_ molecules into value-added chemicals when coupled with renewable electricity sources such as solar, wind, or tidal energy, thereby facilitating the transition from a fossil-fuel-dominated economy to a circular, carbon-neutral economy [9–12].

**Figure 1.**
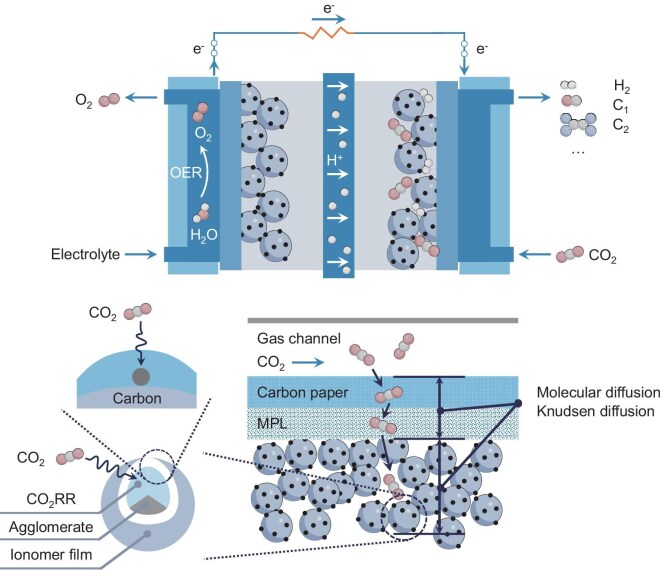
Schematic diagram of an electrolyzer structure with insets illustrating the typical three-phase interface for CO_2_RR.

Nevertheless, the practical implementation of the CO_2_RR faces profound scientific and engineering challenges, particularly in terms of the intrinsic thermodynamic stability and kinetic inertness of CO_2_ molecules. Their linear structure and strong C=O bonds require high activation energies, which result in significant overpotentials during electrocatalysis [13–15]. The reaction mechanism involves multiple proton-coupled electron transfer (PCET) steps and a complex network of possible pathways, leading to a wide range of products, including CO, formate, methane, ethylene, ethanol, and other carbon-based oxygenates and hydrocarbons [16–20]. This product diversity is further complicated by the pervasive hydrogen evolution reaction (HER), which predominates under aqueous conditions and drastically limits the Faradaic efficiency (FE) toward desirable compounds. Consequently, the development of electrocatalysts and electrode systems that exhibit high activity, selectivity, stability, and energy efficiency remains a major barrier to the industrial adoption of the CO_2_RR. Researchers have focused on the design of advanced electrochemical reactors and functional electrode architectures to address these challenges and enable the CO_2_RR at current densities appropriate for commercial applications, often exceeding 300 mA cm^−2^ [21–23]. The electrode is a pivotal component that bridges the catalyst and electrolyzer and governs the mass transport, charge transfer, and reaction distribution. Flow cells and zero-gap membrane electrode assemblies (MEAs) have emerged as leading configurations owing to their ability to manage the triple-phase boundary among the gas (CO_2_), liquid (electrolyte), and solid (catalyst) phases [24–26]. A typical electrolyzer has a multilayer structure and both the anode and cathode consist of gas diffusion layers (GDLs), microporous layers, and catalyst layers with an ion-exchange membrane between them (Fig. 1). During operation, H_2_O is oxidized at the anode, which produces O_2_ via the oxygen evolution reaction (OER) and protons, while CO_2_ diffuses through the GDL to access the catalytic sites and is reduced at the cathode [27–29]. The architecture and composition of the catalyst layer are critical because they directly affect the local environment in which the key interfacial processes occur.

The ionomers play an indispensable and multifunctional role within the catalyst layer, where they serve simultaneously as binders, ion conductors, and microenvironment modifiers [30–32]. As polymeric binders, they enhance the mechanical cohesion of the catalyst layer, improve the adhesion to the substrate, and prevent detachment during long-term operation. They also promote the dispersion of catalyst nanoparticles, mitigate aggregation, and ensure a high density of accessible active sites. Beyond their structural function, ionomers facilitate efficient ion transport and conduct protons, hydroxides, or carbonates between the reaction interface and ion-exchange membrane, thereby maintaining the charge balance during electrolysis [33–35]. By forming a porous yet continuous network throughout the catalyst layer, they further regulate the transport of water, gases, and products and fine-tune the local pH, water activity, and CO_2_ concentration at the electrochemical interface. The chemical nature of ionomers, either cationic or anionic [35–37], affects the reaction selectivity and stability of CO_2_RR electrodes. In this review, we discuss the roles and working principles of ionomers within CO_2_RR catalyst layers, and emphasize the effects of their structural and interfacial properties on the performance and stability of the electrode. We compare the two major classes of ionomers—cationic and anionic—and highlight their respective advantages and limitations under distinct operating conditions. Particular attention is given to ionomer dispersion and its effect on catalyst–ionomer interactions, which govern mass transport, intermediate adsorption, and hydrogen bond networks at the catalyst–electrolyte interface. We conclude by summarizing the critical insights into the effects of ionomers on the CO_2_RR selectivity and efficiency and outline future opportunities for extending ionomer-based design principles to broader electrocatalytic transformations. By synthesizing the available evidence, this review identifies key literature gaps to inform future investigations and outlines design principles for developing next-generation electrodes that enable efficient, selective, and durable CO_2_ conversion. These same principles can extend readily to other electrocatalytic processes.

## TYPES OF IONOMERS

The ionomers used to construct the catalyst layers play a pivotal role in determining the overall performance of the electrochemical devices. Their functionality extends beyond that of simple binding agents, and ionomers have a critical effect on the charge transport, mass transfer, reactant permeability, water management, and interfacial reactivity [38–40]. For these roles to be fulfilled effectively, ionomers must exhibit a specific combination of properties, including high ionic (proton or hydroxide) conductivity, tunable gas permeability, efficient water uptake and transport, strong compatibility with catalyst particles, and robust chemical and electrochemical stability under operating conditions (e.g., against radical attack, pH swings, and applied potentials).

Based on the nature of their functional groups and charge-carrying capacities, ionomers can be classified into two broad categories: proton-exchange (cationic) and anion-exchange (anionic) ionomers (PEIs and AEIs, respectively). Each category imparts distinct physicochemical characteristics to the catalyst layer, thereby affecting the reaction pathways, intermediate stabilization, and product distribution during the CO_2_RR. The selection of cationic or anionic ionomers is not based solely on their conductivity; rather, it is a strategic choice that affects the entire electrode microenvironment.

### PEIs

**Figure 2.**
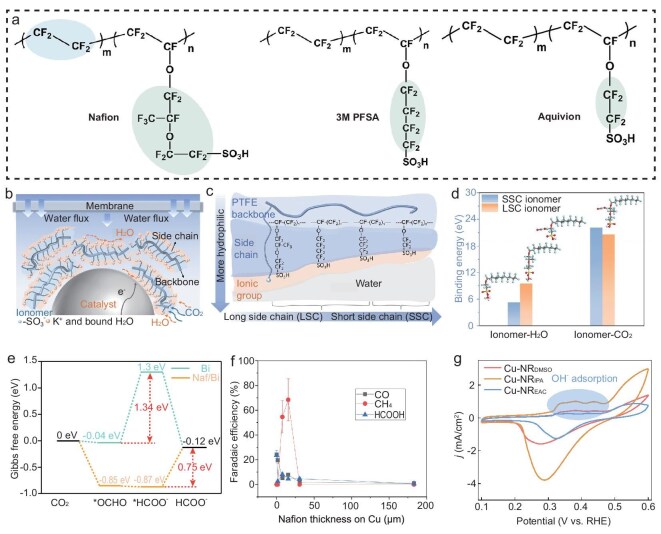
Investigation of PEIs for CO_2_RR. (a) PFSA ionomers with different backbone or side-chain structures. Adapted with permission from [42]. Copyright 2025 Royal Society of Chemistry. (b) The mass transfer diagram of CO_2_ and H_2_O channels at the three-phase interface. (c) Chemical structure and hydropathy properties of PFSA ionomers. (d) Density functional theory calculation results for the binding energies of ionomer-H_2_O and ionomer-CO_2_. Adapted with permission from [43]. Copyright 2025 Royal Society of Chemistry. (e) Gibbs free energies of reaction pathways over Bi and Nafion/Bi. Adapted with permission from [47]. Copyright 2022 Elsevier. (f) FE as a function of Nafion thickness over Cu foil. Adapted with permission from [48]. Copyright 2020 Royal Society of Chemistry. (g) Cyclic voltammetry (CV) curves over various Cu electrodes in 1 M KOH. Adapted with permission from [49]. Copyright 2025 Elsevier.

The most widely used PEIs are perfluorosulfonic acid (PFSA)-based polymers (Fig. 2a). They consist of a hydrophobic polytetrafluoroethylene (PTFE) backbone with periodic perfluoroether side chains that terminate in hydrophilic sulfonic acid groups (–SO_3_H) [41,42]. This architectural motif leads to a microphase separation between the hydrophobic and hydrophilic domains, especially under hydrated conditions, forming interconnected ion-conducting channels that enable efficient proton transport [43] (Fig. 2b and c). The presence of strong C–F bonds and the steric shielding provided by the fluorine atoms endow PFSA with exceptional chemical inertness, hydrophobicity, and stability.

In CO_2_RR systems, Nafion is often used as an ultrathin film (1–10 nm) applied to catalyst surfaces [44]. These films serve as proton conductors and as binders that increase the adhesion of the catalyst to the GDLs and improve the mechanical cohesion of the catalyst layers. However, when confined in such thin layers, Nafion exhibits morphological and transport properties that differ significantly from those of bulk membranes. The distinction between a thin-film ionomer and a bulk membrane is critical. Bulk membranes exhibit substantial water uptake and swelling, which can lead to cathode flooding. In contrast, an ultrathin ionomer layer on a catalyst surface is constrained by the substrate and its nanoscale thickness. The hydrophobic PTFE backbone dominates, while sulfonic acid groups are partially buried, resulting in limited water uptake and negligible swelling. Consequently, the hydrophobic surface repels excess liquid water, maintaining a thin water layer sufficient for proton conduction but insufficient to cause flooding. This demonstrates that a thin Nafion ionomer can suppress HER without the catastrophic flooding expected for a bulk membrane. Restricted polymer chain mobility and interfacial interactions can affect water uptake, reduce proton conductivity, and modify CO_2_ diffusion pathways (Fig. 2d) [45,46]. The intrinsically low free volume of the PTFE backbone further limits the CO_2_ permeability, which may constrain the mass transport at high current densities.

Notably, Nafion plays an active role in modulating the reaction selectivity. When applied to Bi catalysts for CO_2_-to-formate conversion (Fig. 2e), it increases the local proton availability and changes the reaction mechanism, favoring formate desorption over further hydrogenation [47]. When applied to Cu electrodes, the proton flux at the surface can be controlled by varying the thickness of the Nafion layer (Fig. 2f), which can stabilize key intermediates such as *CO and promote its further reduction to methane [48]. Nafion is particularly effective in flow cell configurations where water management is critical. It can trap OH^−^ generated *in situ* (Fig. 2g), optimizing the transport of H_2_O and creating a microenvironment rich in both OH^−^ and free H_2_O at the catalyst interface, which is crucial for achieving a high FE_C2+_ (exceeding 90% at high current densities). Furthermore, the hydrophobic domain of the thin-film Nafion ionomer reduces water activity at the catalyst interface, thereby suppressing the HER without causing electrode flooding [49]. Its functional groups can also interact with CO_2_ via Lewis acid-base interactions, potentially activating CO_2_ molecules and facilitating C–C coupling for C_2+_ products. However, despite these advantages, PFSA ionomers face several practical challenges. For example, their synthesis involves complex and costly fluorination processes; they are prone to swelling, which may block the catalyst pores; their acidic groups may interfere with the catalytic sites; and the presence of fluorine raises environmental and recycling concerns. Moreover, catalyst recovery and ionomer removal after operation remain nontrivial. Research into partially fluorinated or fully hydrocarbon-based analogs seeks to address these issues while retaining the beneficial microphase-separated morphology.

### AEIs

AEIs typically feature aromatic (unsaturated) polymer backbones, such as poly(arylene ether), poly(arylene ether sulfone), poly(arylene ether ketone), polyphenylene, poly(fluorene), poly(benzimidazolium), poly(carbazole), and poly(arylene piperidinium). These aromatic structures offer superior mechanical robustness, thermal stability, and gas-barrier properties compared to their saturated aliphatic counterparts, which are critical for maintaining a stable triple-phase boundary in gas-fed CO_2_ electrolyzers [50–52].

A widely studied commercial AEI is Sustainion, which contains imidazolium functional groups. The imidazolium ring exhibits high affinity for CO_2_, imparting CO_2_ solubilities up to 20 times higher than those in Nafion [53]. This property increases the local CO_2_ concentration and the CO_2_/H_2_O ratio at the catalyst surface, favoring CO_2_RR over HER. Moreover, the cationic groups help buffer the interfacial pH by incorporating *in-situ*-generated bicarbonate ions, creating a stable alkaline environment that promotes C–C coupling.

While aromatic backbones are common, recent advances have demonstrated that saturated backbones can also achieve excellent performance when designed appropriately. For example, polynorbornene-based AEIs [54] (e.g., Pention-D18 functionalized with quaternary ammonium groups) combine high ionic conductivity with minimal swelling. Despite having a fully saturated alkane backbone, these materials enrich OH^−^ near the catalyst surface, increase local pH (up to ∼11.39), suppress HER, and accelerate C_2+_ formation. The anti-swelling property of the polynorbornene backbone allows precise control of water content (2%–7%) in the catalyst layer, with an optimal content of ∼3.5% enhancing proton-electron transfer for C_2+_ products.

*In situ* spectroscopic and computational studies have shown that the hydrophobicity of the ionomer side chains is a critical descriptor. Hydrophobic AEIs [e.g., Pention ionomers with –C_4_H_8_N^+^(CH_3_)_3_ side chains] strengthen *CO adsorption on Cu and slow its desorption via an electrochemical kinetic retardation mechanism, thereby increasing surface *CO coverage and the probability of C–C coupling. This leads to higher C_2+_ selectivity compared to ionomers with similar or lower hydrophobicity. Hydrophobic AEIs induce a less compact and less hydrated electrical double layer, favoring the stabilization of CO-derived intermediates and enhancing selectivity toward ethylene and ethanol. In contrast, more hydrophilic ionomers (e.g., poly(terphenyl piperidinium), PTP) tend to promote HER and formate production [55].

In summary, PEIs and AEIs both offer distinct advantages in the CO_2_RR catalyst layers, and the choice of ionomer is tailored to the catalyst, desired products, and operating conditions (e.g., flow cells vs. MEAs). Although PFSA ionomers involve complex fluorination processes, AEIs are often significantly more expensive (PFSA: ∼$5–20 USD g^−1^; AEIs: ∼$50–500 USD g^−1^ for research-grade materials). Ongoing research aims to develop non-fluorinated, low-cost, and highly durable ionomers that provide precise control over the electrode microenvironment for efficient and selective CO_2_RR, with advanced designs such as bilayer ionomer structures showing great promise by combining the benefits of both types of ionomers. For example, Kim *et al*. achieved 90% FE for C_2+_ products with only 4% H_2_ evolution and a 250% enhancement in C_2+_ production compared to static electrolysis on bare Cu using a Nafion/Sustainion bilayer coating [53].

## DISPERSION OF IONOMERS

The pursuit of an efficient and selective electrochemical CO_2_RR that can be used to produce value-added chemicals and fuels necessitates meticulous engineering of the architecture of the catalyst layers. This microstructure, which ultimately dictates the performance, mass transport properties, and durability of the system, is mainly fabricated during the preparation of the catalyst ink, which is a colloidal suspension consisting of the electrocatalyst, an ionomeric binder, and a dispersion solvent. The ionomer, typically a PFSA polymer such as Nafion or its derivatives, conducts protons to active sites and acts as a binder to ensure the mechanical integrity of the system. However, an optimal ionomer distribution requires a delicate balance. Low ionomer contents lead to poor ionic connectivity and incomplete catalyst utilization, whereas excessive coverage creates transport barriers for CO_2_ diffusion, promotes a parasitic HER through water trapping, and can lead to electrode flooding. Consequently, the formulation of the catalyst ink, and specifically, the selection and processing of the dispersion solvent, must be considered as part of a materials processing strategy that can control the nanoscale conformation, mesoscale aggregation, and ultimate spatial distribution of the ionomer within the dried catalyst layer, thereby controlling the electrochemical microenvironment of the electrode and the catalytic output.

### Solvent-directed ionomer conformation and aggregation in solution

The physicochemical properties of the dispersion solvent, particularly its dielectric constant (*ε*), viscosity, and specific chemical affinity for the ionomer hydrophobic backbone compared to the hydrophilic functional groups, exert a considerable effect on the ionomer solvation state and supramolecular structure in the ink. PFSA ionomers, which are characterized by a PTFE backbone and pendant sulfonic acid-terminated side chains, exhibit a strong tendency for microphase separation. In solution, this manifests as the formation of aggregates, the size and morphology of which are highly sensitive to the solvent environment.

**Figure 3.**
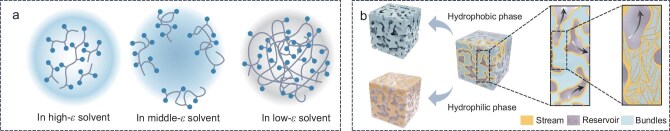
Dispersion of ionomers. (a) The morphology of Nafion in different dispersion solvents. (b) 3D scheme of the stream-reservoir model.

The morphology of the ionomer depends strongly on the solvent environment [56]. The *ε* of the solvent affects its ability to solvate ionic polymers. Specifically, when *ε* > 10, 3 < *ε* < 10, and *ε* < 3, PFSA ionomers exist predominantly as molecularly dissolved species, colloidal aggregates, or precipitates, respectively [57]. For example, in ethyl acetate (*ε* = 6.0), PFSA readily forms large agglomerates and fails to form a stable dispersion, whereas in dimethyl sulfoxide (*ε* = 48.9), rapid phase separation occurs in the catalyst ink (Fig. 3a). Advanced characterization techniques such as dynamic light scattering (DLS), small-angle X-ray scattering (SAXS), and cryoelectron transmission electron microscopy (cryo-TEM) have been used to quantitatively elucidate this relationship [46].

Studies using binary mixtures of dipropylene glycol (DPG) and water have demonstrated that the hydrodynamic diameter *D*_h_ of Nafion aggregates can be tuned systematically by several orders of magnitude [58]. In pure water (high *ε*, strong preference for the –SO_3_H groups), large, micron-scale aggregates (*D*_h_ ≈ 2 μm) form owing to dominant hydrophobic interactions between the backbones. By contrast, in pure DPG (lower *ε*, high affinity for the PTFE backbone), the ionomers are more dispersed and form much smaller nanoscale aggregates (*D*_h_ ≈ 8 nm). A 50:50 wt% mixture of DPG and water yielded an intermediate size of ∼50 nm. Although smaller ionomer aggregates (e.g., ∼8 nm in pure DPG) produce a more uniform ionomer distribution, the optimal power performance is achieved at an intermediate aggregate size (∼50 nm in 50 wt% DPG) that matches the size of the primary catalyst aggregates, thereby balancing proton conductivity and gas transport. This exponential reduction in the aggregate size as the DPG content increased was attributed to the superior solvating power of DPG for the fluorocarbon backbone, which effectively screened the hydrophobic driving force for aggregation. Molecular dynamics simulations provided atomistic insights into these interactions [59]. They revealed that the (–CF_2_–CF_2_–) backbones were predominantly solvated by solvent molecules through hydrophobic interactions, while the –SO_3_H was strongly coordinated by water molecules via electrostatic forces. This differential solvation explains the conformational changes [60]. The solvation energy becomes increasingly negative (more favorable) in the order water (−147 ± 9 kcal mol^−1^) < DPG/water mixture (−216 ± 21 kcal mol^−1^) < DPG (−444 ± 9 kcal mol^−1^), confirming that DPG promotes a more extended and well-dispersed ionomer state. Consequently, the radius of gyration follows the opposite trend, and it is larger in DPG (17.2 ± 1.0 Å) than in water (13.6 ± 0.4 Å), which indicates that the chain conformation collapses more in aqueous environments.

The implications of this solvent-controlled aggregation extend directly to the catalyst ink rheology and stability. Inks formulated with solvents that induce large ionomer aggregates (e.g., water-rich ones) tend to have broader aggregate size distributions and exhibit less stable colloidal behavior. The zeta potential of the catalyst particles, a key indicator of colloidal stability, is also affected by the solvent [61]. In general, increasing the solvent dielectric constant or ionomer content increases the zeta potential of the catalyst particles, which increases the electrostatic repulsion and reduces the size of the catalyst agglomerates in the ink. However, beyond an optimal ionomer mass fraction (∼2.5 wt%), the excess counter-ions released from the ionomer induce a charge screening effect that compresses the electrical double layer, reduces the absolute zeta potential, and may lead to re-agglomeration of the catalyst.

### From ink to electrode: translating the dispersion structure to the catalyst layer microenvironment

The nanostructure imprinted on the ionomer in the dispersion is largely retained upon drying, which determines the microstructure and resulting performance of the catalyst layer. The size of the ionomer aggregates in the ink dictates their packing and distribution around the catalyst particles. The guiding principle is that ionomer aggregates comparable in size to the primary catalyst particles form the most homogeneous and efficient networks, a concept initially established in fuel cell research and subsequently validated in CO_2_RR systems, where tuning ionomer aggregate size via solvent selection has been shown to significantly enhance CO_2_RR activity.

For example, dispersions in pure DPG yield ∼20 nm ionomer aggregates, which are comparable to the primary particles in Pt/C catalysts [56]. Upon coating and drying, this forms thin, uniform films that conformally cover the catalyst and carbon support, thereby establishing a continuous proton-conducting network and minimizing pore blockage (Fig. 3b). By contrast, water-based dispersions with micron-scale aggregates produce highly heterogeneous films upon drying, leading to regions with thick ionomer layers that block pores and hinder gas transport, as well as regions where the catalyst is exposed that have limited proton access.

The nanoscale architecture directly determines the mesoscale properties of the catalyst layer. Nitrogen physisorption measurements show that the mesopore volume decreases as the dispersion solvent switches from water to DPG, indicating that the finer, nanodispersed ionomers from the DPG-rich inks penetrate and occupy the complex pore network within and between the catalyst agglomerates more effectively than the coarser ionomers from the water-rich inks. Moreover, the resulting catalyst layer is more compact and mechanically robust, with reduced overall porosity, and this densification often establishes a more continuous and optimal triple-phase boundary [62].

The choice of solvent also affects electrode wetting and the local electrochemical environment, which is critical for gas-fed CO_2_RR electrodes operating at delicate gas–liquid–solid interfaces. Solvents exhibiting a higher affinity for the ionomer backbone, such as acetone or DPG, facilitate the formation of more hydrophobic catalyst layers. Studies on Cu-based gas diffusion electrodes (GDEs) have demonstrated that using acetone rather than methanol as the dispersion solvent yields a more continuous and hydrophobic catalyst layer. This is because high-affinity solvents solvate the hydrophobic PTFE backbone of the ionomer, causing it to adopt an extended backbone conformation in solution. Upon drying, this conformation is retained, enriching the hydrophobic backbone at the film surface and reducing surface energy, thereby yielding a more hydrophobic catalyst layer [63]. The increased hydrophobicity mitigates water flooding and preserves efficient gas transport pathways, thereby increasing the CO_2_ mass transfer and maintaining an optimal local CO_2_/H_2_O ratio at the catalyst surface [64]. This environment promotes C–C coupling, thus improving the selectivity toward C_2+_ products over the competing HER or C_1_ pathways.

Moreover, the solvent affects the acidic characteristics of the ionomer and the accessibility of the ion-exchange sites in the final layer. The acidity of the ionomer dispersions increases (lower pH) as the water content increases [65], which reflects the more effective dissociation of sulfonic acid protons. However, the fraction of accessible protons relative to the theoretical ion-exchange capacity depends on the ionomer chemistry and dispersion state, which highlights persistent differences in the accessibility of the acid groups in the films.

The performance at the high current densities required for industrial applications is particularly sensitive to mass transport, which is governed by the structure of the catalyst layers. Electrodes fabricated from inks designed to yield a homogeneous ionomer distribution and maintain porosity demonstrate lower operational overpotentials and superior stability. For example, a study using an ionomer preconfinement strategy during Ag catalyst synthesis created a GDE with a uniform ionomer distribution and efficient mass transfer pathways [66]. This electrode achieved an FE_CO_ of >90% at current densities of up to 600 mA cm^−2^ and operated stably for more than 220 h, even with low-concentration CO_2_ feeds.

The mechanical integrity of catalyst layers, which is crucial for their long-term durability, is also affected by the ink solvent. Solvents that promote stronger adhesion between the ionomer and catalyst particles, often those with higher dielectric constants (e.g., *ε* = 70 or 55) [61], result in catalyst layers with a higher fracture toughness. This reduces the propensity for crack formation and delamination during operation, thereby increasing the operational lifespan of the electrode.

In summary, the dispersion solvent is not an inert component of the catalyst ink formulation; instead, it is a key processing parameter that governs the ionomer conformation, aggregation, and spatial distribution within the catalyst layer. By judiciously selecting solvents based on their dielectric properties, polymer affinity, and effects on nano- and mesoscale structures, catalyst layers can be engineered to maximize the ionic conductivity, mass transport, and hydrophobicity. Such control over the electrochemical microenvironment provides a critical pathway for achieving high selectivity, efficiency, and stability in the CO_2_RR, thereby advancing these systems toward practical applications.

## EFFECTS OF THE IONOMER CONFIGURATION ON THE CO_2_RR SELECTIVITY AND REACTION PATHWAYS

The molecular architecture of the ionomers represents a critical design parameter that exerts profound and multifaceted effects on the fundamental reaction pathways of the electrochemical CO_2_RR. By strategically manipulating the chemical structure of the ionomer, including the identity of the functional groups, length and flexibility of the side chains, hydrophobicity/hydrophilicity balance of the backbone, and the overall ionic character (cationic vs. anionic), the catalytic microenvironment can be engineered precisely at the molecular level. This engineering capability enables remarkable control over the reaction selectivity, enhances the efficiency, and suppresses competing pathways such as the HER.

The ionomer configuration primarily manifests its effects through three interconnected mechanisms: sophisticated regulation of the mass transfer phenomena, subtle modulation of the key intermediate adsorption energetics, and restructuring of the interfacial hydrogen bond network. Each of these mechanisms makes a unique contribution to the ultimate product distribution and overall system performance during the CO_2_RR. The effect of these structural modifications has been shown across multiple studies, where seemingly minor changes in the side chain length or functional group yield dramatic, nonlinear improvements in the product selectivity and operational stability, underscoring the importance of ionomers as a dynamic, active component rather than a passive binder.

### Mass transfer

**Figure 4.**
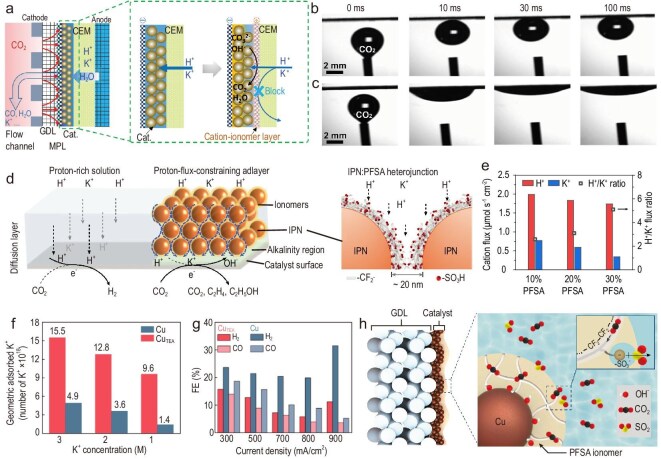
The influence of ionomer configuration on the mass transfer. (a) Schematic illustration of mass transport inside a cathodic chamber with a cation-ionomer layer. Cat., catalyst. Adapted with permission from [68]. Copyright 2024 Royal Society of Chemistry. (b–c) Real-time CO_2_ bubble transport behaviors for GDE (b) and GDE@Sustainion (c). Adapted with permission from [70]. Copyright 2025 Springer Nature. (d) Schematics of interfacial reactions and proton transport near the catalyst surface. IPN, insulating polymer nanoparticles. (e) The apparent cation fluxes passing through various COF: PFSA adlayers and the corresponding H^+^/K^+^ flux ratios. Adapted with permission from [72]. Copyright 2023 Springer Nature. (f) Adsorbed K^+^ on the Cu and Cu_TEA_ surfaces in KCl solutions with different concentrations. (g) FE_H2_ and FE_CO_ vs. current density over Cu and Cu_TEA_ electrode. Adapted with permission from [73]. Copyright 2025 American Chemical Society. (h) Schematic of a planar PFSA@Cu/PTFE heterojunction with hydrophilic and hydrophobic characteristics provided by –SO_3_^−^ and –CF_2_ functionalities for interacting with SO_2_ and facilitating CO_2_ transport, respectively. Adapted with permission from [74]. Copyright 2025 Springer Nature.

The configuration of the ionomer serves as a fundamental determinant of the mass transport properties within the catalyst layer, which governs the local concentrations and the availability of reactants (CO_2_ and H_2_O) and products. The hydrophobicity of the ionomer backbone and side chains is a pivotal factor affecting the mass transfer characteristics. Hydrophobic ionomers, exemplified by those incorporating long alkyl chains such as C_16_H_33_-side chains [67], actively create a water-depleted environment in proximity to the catalyst surface. This engineered hydrophobic microenvironment performs multiple crucial functions. For example, it effectively repels excess water molecules, thereby mitigating the electrode flooding phenomena, and greatly reduces the local availability of protons that would otherwise participate in the competing HER [68]. In contrast, ionomers with pronounced hydrophilicity tend to exhibit contrasting effects. Cation exchange membrane (CEM) preferentially facilitate proton (H^+^) transport (Fig. 4a), which can potentially acidify the local catalytic environment and promote the HER unless carefully balanced with other design parameters, as noted by Peerlings *et al*. [69] and Chang *et al*. [55].

The concentration and retention time of gaseous CO_2_ are essential for achieving the desired CO_2_RR pathways. Qin *et al*. reported that coating a Cu catalyst surface with imidazolium-based anion-conducting Sustainion enabled the enrichment of diluted CO_2_ to establish a high local CO_2_ concentration [70], and simultaneously hindered proton transport to suppress the HER. This ionomer layer created a well-defined gas–liquid–solid triple-phase boundary, thereby facilitating the efficient electroreduction of low-concentration CO_2_ in acidic media (Fig. 4b and c).

Advanced ionomer architectures incorporating porous frameworks, such as the covalent organic framework (COF) described by Fang *et al*. [71], provide additional mass transfer advantages by creating well-defined nanochannels that enhance CO_2_ accessibility and facilitate the targeted enrichment of specific cations at the catalyst surface, thereby offering unprecedented control over reactant delivery and product distribution. Zhao *et al*. [72] demonstrated the effectiveness of a nanoconfinement strategy and achieved remarkable C_2+_ selectivity (>75%) and operational stability (>30 h) through ionomer preconfinement, which established uniformly distributed mass-transfer pathways. In their system, a COF was intimately interfaced with a PFSA ionomer, leading to the reorganization of the ionomer network into well-defined hydrophilic–hydrophobic nanochannels that precisely regulate ionic and molecular transport (Fig. 4d). This confined morphology suppresses excessive proton flux to the catalyst surface, thereby mitigating the competing HER, while simultaneously enriching K^+^ cations within the hydrophilic ionic domains adjacent to the electrode (Fig. 4e). Moreover, the preconfined ionomer architecture narrows the proton-conducting pathway, spatially constraining proton delivery such that key *CO intermediates are more likely to undergo coupling rather than premature hydrogenation.

Bicarbonate/carbonate ions (HCO_3_^−^/CO_3_^2−^) can also be balanced with K^+^, which helps maintain a more alkaline local pH environment that favors CO_2_RR pathways requiring such conditions, especially those leading to C_2+_ products. Recent studies have revealed that the configuration of ionomers, particularly the degree of microphase separation between hydrophobic PTFE backbones and hydrophilic sulfonic acid domains, plays a decisive role in regulating the interfacial distribution, hydration, and enrichment of K^+^ cations. Enhanced microphase separation, as achieved by reorganizing the Nafion network [73], increases the hydrophobicity of the catalyst layer and isolates sulfonic domains, thereby reducing the density of interfacial water and facilitating K^+^ dehydration. Molecular dynamics simulations and *in-situ* spectroscopic characterizations confirm that this reconfiguration mediated by tetraethylammonium (TEA) strengthens hydrogen bonding between sulfonic acid groups and water molecules, effectively removing the hydration shell around K^+^ while simultaneously enriching it at the catalyst surface through electrostatic attraction (Fig. 4f). The dehydrated K^+^ species exhibit stronger electrostatic coupling with adsorbed *CO intermediates, lowering the energy barrier for C–C coupling and favoring C_2_H_4_ formation. A well-optimized microphase-separated morphology not only establishes a hydrophobic yet ionically conductive microenvironment that suppresses parasitic HER (Fig. 4g) but also promotes *CO coverage and multicarbon product selectivity, demonstrating that the nanoconfiguration of Nafion is a powerful lever for tuning the cationic microenvironment in CO_2_RR.

In addition to modulating H_2_O, CO_2_, and K^+^ transport, ionomers can regulate impurities in the gas phase. For example, SO_2_ in flue gas can poison catalysts and degrade the material performance. The polar HSO_3_ groups of the PFSA ionomer in Nafion interact strongly with SO_2_, suppressing SO_2_ transport and resulting in high SO_2_ solubility (∼30 times that of nonpolar CO_2_) and a low diffusion rate (∼one-fifth of that of CO_2_). By contrast, the hydrophobic PTFE backbone provides rapid transport channels for nonpolar CO_2_, thereby enhancing the CO_2_/SO_2_ mass flux ratio at the Cu surface (Fig. 4h). Consequently, PFSA@Cu/PTFE electrodes exhibit excellent SO_2_ tolerance at high current densities and enable energy-efficient operation [74].

### Adsorption of intermediates

**Figure 5.**
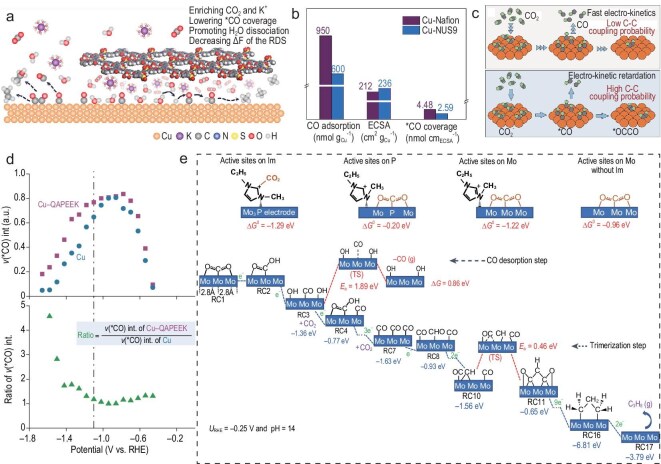
The influence of ionomer configuration on the adsorption of intermediates. (a) Schematic illustration of COF ionomer. (b) Electrochemical characterization of Cu-Nafion and Cu-NUS9. Adapted with permission from [75]. Copyright 2025 American Chemical Society. (c) Schematic illustrations of enhancing C_2+_ selectivity by electro-kinetic retardation. Adapted with permission from [77]. Copyright 2023 Wiley-VCH Verlag. (d) The *ν*(*CO) peak intensity and ratio vs. potential. Adapted with permission from [78]. Copyright 2022 Springer Nature. (e) Gibbs free energy profile for CO_2_RR to C_3_H_8_ over ImF-Mo_3_P. Adapted with permission from [79]. Copyright 2023 Springer Nature.

Ionomers exert significant effects on the adsorption strength and surface coverage of critical reaction intermediates through direct chemical interactions and indirect field effects, thereby affecting the reaction energy landscape and ultimately determining the product selectivity. This multifaceted interaction mechanism operates through several distinct pathways that collectively determine the catalytic outcomes. The charged functional groups within the ionomer structures create substantial electrostatic fields (manifesting as Donnan potentials) that selectively concentrate specific ionic species near the catalyst surface. The 2D sulfonated COF nanosheets ionomer (NUS9) provides an exemplary demonstration of this principle (Fig. 5a) [75]. This targeted cationic enrichment has been corroborated through combined computational and experimental approaches, showing a direct correlation with an increase of electrochemical surface area (ECSA) and a reduction in the *CO surface coverage. This is a crucial mechanistic step that shifts selectivity away from C_2+_ products and toward methane formation, illustrating how ionomer-induced field effects can redirect reaction pathways (Fig. 5b). Similarly, Heim *et al*. [76] demonstrated that tuning the K^+^ content within polystyrene-based ionomers significantly increased the C_2+_ partial current density up to 225 mA cm^−2^. Their molecular dynamics simulations revealed the effects of stabilizing intermediates within the ionomer microenvironment. Zhuansun *et al*. [77] proposed a water uptake strategy in which a hydrophobicity-induced electrokinetic retardation effect significantly prolonged the residence time of *CO intermediates on Cu surfaces (Fig. 5c), greatly increasing the C–C coupling probability and generating a remarkable FE_C2+_ of ∼70% in pure water and 90% in bicarbonate electrolyte.

Beyond electrostatic effects, specific functional groups strategically incorporated into ionomer architectures can engage in precise chemical interactions with the reaction intermediates. The bifunctional ionomer quaternary ammonium–functionalized poly(ether ether ketone), which incorporates strategically positioned carbonyl groups within its polymeric backbone [78], was designed to activate CO_2_ molecules through specific chemical interactions. When coated on Cu foil electrodes, this functionalized ionomer significantly enhances ethylene selectivity, demonstrating how a targeted molecular design can stabilize specific transition states or intermediates to control the CO_2_RR pathways (Fig. 5d). Similarly, imidazolium-functionalized ionomers (Im), which are integrated with Mo_3_P or MoP catalysts, can effectively modulate the electronic properties of the surface metal atoms through coordination interactions. This electronic modification phenomenon increases the adsorption energy of carbon-based intermediates, thereby facilitating specific reaction pathways toward targeted products such as propane or ethanol (Fig. 5e), as documented by Esmaeilirad *et al*. [79,80] The considerable differences in the product distribution observed when using various ionomers^55^ (Nafion favoring C_2+_, XA-9 favoring CO, and PTP favoring H_2_ and formate production) on identical Cu catalyst systems further underscore the ability of the ionomer configuration to control the intermediate binding energetics and consequently, the patterns of catalytic selectivity through sophisticated molecular-level interactions. This adsorption modulation extends beyond carbon intermediates to include oxygenated species.

### Hydrogen bonding network

**Figure 6.**
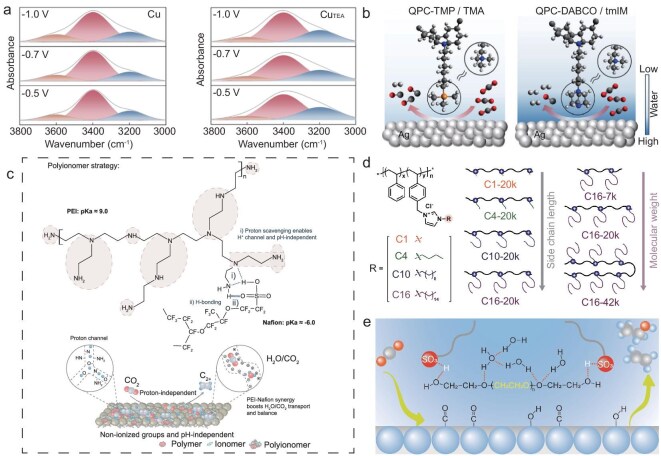
The influence of ionomer configuration on the hydrogen bond network on the electrode surface. (a) *In situ* ATR-SEIRAS of interfacial water. The OH stretching bands are attributed to 4H–H_2_O (blue), 2H–H_2_O (red) and K^+^–H_2_O (orange) as determined by Gaussian fitting. Adapted with permission from [73]. Copyright 2025 American Chemical Society. (b) Schematic illustration of ionomer environment on Ag surface. Adapted with permission from [85]. Copyright 2025 American Chemical Society. (c) The optimal structure–property function of the polyionomer across a wide pH range governed by its chemical structure, molecular orientation, and p*K*a values. Adapted with permission from [87]. Copyright 2025 American Chemical Society. (d) Chemical structures of 1-*n*-alkylimidazolium ionomers with n-alkyl side chains of varying lengths. Adapted with permission from [86]. Copyright 2025 American Chemical Society. (e) Schematic illustration of the surface configuration of species on Cu_PEG_ surfaces. Adapted with permission from [88]. Copyright 2025 Wiley-VCH Verlag.

The structure and dynamics of the interfacial water network, which serves as an essential proton donor for all the PCET steps in the CO_2_RR and HER, can be controlled by the molecular configuration of the ionomer [81,82]. The chemical nature of the functional groups of the ionomer dictates how it interacts with water molecules [83], which determines the critical proportion of free water versus strongly or weakly hydrogen-bonded water molecules within the interfacial region by attenuated total reflection surface-enhanced infrared absorption spectroscopy (ATR-SEIRAS) (Fig. 6a). This deliberate structuring of the water organization has direct and dramatic consequences for the reaction kinetics and selectivity.

Advanced *operando* spectroscopic studies have revealed that interfacial water exhibits distinct structural dynamics compared to bulk water [84]. Under applied potential, water molecules near the electrode reorganize into a more ordered hydrogen-bond network, accompanied by a shift of O–H stretching bands toward higher frequencies, indicative of strengthened hydrogen bonding and reduced configurational entropy. Such ordering arises from the strong interfacial electric field, which aligns water dipoles and facilitates the formation of extended hydrogen-bonded domains. Within CO_2_RR catalyst layers, this structured water network plays a pivotal role in mediating proton transfer and stabilizing reaction intermediates. The ionomer microenvironment further refines this organization by confining water within nanoscale domains, adjusting its activity and mobility. Consequently, the hydrogen-bond network at the electrochemical interface serves as a dynamic medium that bridges molecular structure, charge transport, and catalytic selectivity. For example, specific ionomers, including polycarbazole-based anion-exchange-trimethylammonium and polycarbazole-based anion-exchange-trimethyl phosphonium, induce the formation of a high fraction of strongly hydrogen-bonded water, characterized by low Stark tuning slopes [85]. This ‘rigid’ or ‘structured’ water configuration proves less energetically favorable for proton dissociation and transport processes, which constitute the rate-determining steps for the HER (Fig. 6b). In contrast, ionomers composed of 1,4-diazabicyclo [2.2.2] octane or trimethyl imidazole (tmIM) are expected to promote the HER. The imidazolium ring in tmIM can directly catalyze the reduction of bicarbonate intermediates, thereby accelerating the HER. Consequently, these engineered ionomers effectively suppress the HER kinetics and maintain the necessary proton availability for the CO_2_RR. This finding has been reported consistently by Park *et al*. [86].

By contrast, ionomers that promote a more disordered free water network facilitate rapid proton transfer, thereby accelerating competing HER pathways. This mechanistic understanding has been confirmed through kinetic isotope effect investigations. The observed reaction rates in D_2_O environments were consistently lower than those in H_2_O environments across all tested ionomers; however, the decrease was greatest for ionomers that created the most rigid water networks (particularly tmIM), which indicates a substantially more sluggish protonation step during the CO_2_RR under these engineered conditions [85]. The ability to precisely engineer the hydrogen bond network through a strategic ionomer design provides a powerful methodology for achieving selective proton transfer control.

By favoring proton delivery to specific CO_2_RR intermediates (e.g., enhancing *CO protonation to *CHO) over direct H^+^ combination (which leads to the HER), advanced ionomers such as QPC-TMP can effectively decouple the catalytic interface from the properties of the bulk electrolyte. This decoupling phenomenon allows high CO_2_RR selectivity (Fig. 6c) to be maintained, even under inherently acidic conditions, as demonstrated by the acidic media operation reported by Polesso *et al*. [87] This fundamental principle explains the superior performance of hydrophobic ionomers with extended alkyl chains (e.g., C16-20k) in acidic operational environments [86] because their molecular architecture limits the availability of interfacial water (Fig. 6d) and sustains an optimized local water structure that preferentially supports selective CO_2_ reduction reaction pathways over competing hydrogen evolution. This hydrogen bond engineering approach extends beyond traditional ionomers. Innovative systems, such as the polyethylene glycol (PEG)-modified electrodes described by Wang *et al*. [88], actively disrupt the hydrogen-bonding network of interfacial water to modulate proton availability and reaction selectivity. *Operando* ATR-SEIRAS and hydrogen-1 nuclear magnetic resonance spectroscopy (^1^H NMR) analyses revealed that PEG interacts strongly with water molecules through both –OH···O and O···H–O hydrogen bonds, forming stable PEG–H_2_O complexes that outcompete water–water interactions. This reconstruction of the hydrogen-bond network reduces the fraction of strongly hydrogen-bonded water while increasing weakly bonded species, effectively lowering the activity and mobility of surface water. Molecular dynamics simulations further showed that PEG incorporation decreases the total number of water–water hydrogen bonds and increases the distance between interfacial water and the electrode surface (Fig. 6e). Such reorganization weakens water dissociation and limits the generation of *H intermediates, thereby suppressing HER. Concurrently, PEG–Nafion hydrogen bonding relaxes the ionomer conformation, exposing additional Cu active sites and enhancing the co-adsorption of *CO and *OH intermediates, which collectively promote C–C coupling toward C_2+_ products. Although PEG disrupts the hydrogen-bond network while Jung *et al*. [85] strengthened it, both strategies suppress HER and enhance CO_2_RR by targeting different levels of water organization, intramolecular bonding strength versus overall network topology.

It is important to acknowledge the experimental challenges in probing the ionomer/water interface. *Operando* techniques such as ATR-SEIRAS and Raman spectroscopy cannot easily distinguish water molecules directly associated with the ionomer from those in the bulk electrolyte, due to overlapping O–H bands, the nanoscale thickness of the ionomer layer, and the exponential decay of surface enhancement. Therefore, the proposed mechanism of HER suppression via rigid hydrogen-bond networks should be viewed as a working hypothesis consistent with current data, pending future validation by more interface-sensitive methods.

By controlling the configuration, ionomers can transcend their traditional roles as binders or proton conductors, and they emerge as an active designer component that can decisively dictate the CO_2_RR pathway through interconnected physical and chemical mechanisms. By strategically engineering the molecular structure of the ionomer to manage mass transfer phenomena, manipulate intermediate adsorption energetics, and control the hydrogen bond network architecture, researchers can precisely tailor the catalytic microenvironment to improve reaction selectivity, enhance efficiency metrics, and unlock novel pathways for the sustainable electrochemical conversion of CO_2_ into valuable fuels and chemical feedstocks. The sophisticated interplay among mass transfer, adsorption control, and hydrogen bond engineering represents the frontier of ionomer design in advanced CO_2_ electrolysis systems.

While the mechanistic insights presented in this section are largely derived from Cu-based CO_2_RR systems, the underlying physicochemical principles, mass transport regulation, electrostatic and chemical modulation of intermediate adsorption, and hydrogen-bond network engineering, are broadly applicable to other catalyst classes (e.g., Ag, Bi, Sn, Pd). The role of ionomer as a microenvironment modifier is decoupled from the intrinsic activity of the catalyst to a significant extent. Nevertheless, the product outcome of ionomer engineering is inherently system-dependent. Over Cu, enhanced *CO coverage promotes C–C coupling, whereas over Ag it primarily improves CO selectivity [89], and over Bi or Sn it favors formate production or alters the proton dependence of the reduction pathway [62,90]. Future studies should systematically compare ionomer effects across different catalyst families to establish a unified design framework. In the present review, we focus on Cu as a model system for its rich product spectrum, but we emphasize that the strategies discussed, hydrophobicity tuning, side-chain engineering, and hydrogen-bond control, are general tools for tailoring the electrocatalytic microenvironment.

## SUMMARY AND OUTLOOK

The electrochemical CO_2_RR is a transformative technology for closing the carbon cycle and producing sustainable fuels and chemicals. Significant progress has been made, particularly in terms of enhancing the performance at current densities relevant to industrial applications. A critical, yet historically underexplored, factor in this advancement is the pivotal role of the catalytic microenvironment, which can be engineered precisely through the strategic application of ionomers. The studies discussed in this review highlight the fact that ionomers are far more abundant than inert binders. Moreover, they are dynamic components that decisively govern mass transport, local concentrations of reactants and ions, interfacial water networks, and intermediate adsorption energetics.

The documented studies revealed several key design principles. A uniform ionomer distribution, achieved through innovative preconfinement methods, creates efficient mass transfer pathways for ions and CO_2_, which enables a high FE to be achieved across a wide current density range and produces exceptional operational stability. Furthermore, the molecular architecture of the ionomer, particularly the chemical identity of the functional groups on the side chain (e.g., trimethylphosphonium vs. imidazolium), has proven to be a powerful descriptor of performance. These modifications dictate the physicochemical properties, such as the water uptake, ion conductivity, and CO_2_ permeability, which in turn control the interfacial water environment. Ionomers that foster a high fraction of strongly hydrogen-bonded water with low Stark tuning slopes effectively suppress the HER and promote selective proton transfer in the CO_2_RR. This principle holds, even under challenging conditions such as diluted CO_2_ feeds and elevated temperatures. The concept of tuning the hydrophobicity via elongated alkyl side chains has emerged as another crucial strategy for effectively modulating the water activity and cation hydration at the catalyst surface to sustain a favorable local pH, thereby decoupling the catalyst microenvironment from the bulk electrolyte conditions, which are often deleterious, especially in cation exchange membrane configurations.

Looking forward, several challenges and opportunities define this research trajectory. Although cationic Nafion remains prevalent, its propensity to enrich K^+^ and precipitate carbonates, leading to flooding and performance decay, necessitates the development of novel ionomeric materials. Promising candidates include polycarbazole-based AEIs and nanostructured ionic COFs, which offer precise control over the porosity and charge distribution to enhance the CO_2_ concentration and stabilize key intermediates. Significantly, we must deepen our fundamental understanding of the nanoscale distribution and morphology of the ionomers within the catalyst layer. The self-assembly process during ink deposition is critically affected by the properties of the dispersion solvent, which dictate the final nanostructure of the ionomer, and consequently, its protonic and gas transport characteristics. Bridging this gap in our knowledge requires the development and application of advanced *in situ* diagnostic techniques, such as three-dimensional imaging and *operando* spectroscopy, to probe the catalyst–ionomer interface under realistic operating conditions.

Ultimately, the ionomer must not be considered as an isolated component; instead, it is an integral part of a synergistic catalyst–ionomer system. Future research should focus on the co-design of catalysts and ionomers to create robust, well-defined interfaces that maximize activity, selectivity, and longevity. By addressing these multifaceted challenges through innovative material synthesis, sophisticated characterization, and holistic electrode design, the scientific community can accelerate the development of efficient, stable, and commercially viable CO_2_ electrolyzers, turning the promise of carbon-neutral fuels and chemicals into a practical reality.
